# Eye-Lashes

**Published:** 1880-08

**Authors:** 


### EYE-LASHES.

In Circassia and neighboring countries, the eye-lashes of children are clipped with scissors, at their extreme points, while asleep, every six weeks, giving them in time a beautiful gloss and curve, besides adding to their length and thickness.

